# When the Sad Past Is Left: The Mental Metaphors Between Time, Valence, and Space

**DOI:** 10.3389/fpsyg.2018.01019

**Published:** 2018-06-28

**Authors:** Nicolas Spatola, Julio Santiago, Brice Beffara, Martial Mermillod, Ludovic Ferrand, Marc Ouellet

**Affiliations:** ^1^Université Clermont Auvergne, CNRS, LAPSCO, Clermont-Ferrand, France; ^2^Department of Experimental Psychology, University of Granada, Granada, Spain; ^3^Center for Research on Mind, Brain, and Behavior (CIMCYC), University of Granada, Granada, Spain; ^4^Univ. Grenoble Alpes, CNRS, LPNC, Grenoble, France; ^5^Univ. Grenoble Alpes, LJK, Grenoble, France; ^6^Psychological Sciences Research Institute, Université Catholique de Louvain, Louvain-la-Neuve, Belgium

**Keywords:** mental metaphor, conceptual metaphor, coherent working models, time, valence, space

## Abstract

A mental metaphor is a strategy that consists of completing the representation of a concept with structural components of a correlating concept. Three issues were addressed here to deepen our understanding of this mechanism: the use of mental metaphors between abstract concepts, the simultaneous activation of multiple mental metaphors and the importance of the focus of attention on the relevant dimensions of a mental metaphor. In two experiments, participants made temporal or valence judgments (with their left or right hand) on verbs with a negative or positive meaning and conjugated in the past or future form, allowing for the simultaneous activation of the “time is space”, “valence is space,” and “time is valence” mental metaphors. Left-past/right-future and left-negative/right-positive congruency effects were found, and these effects were greater in the temporal and valence judgment tasks, respectively, demonstrating the importance of attentional cuing. Simultaneously, a congruency effect between the abstract concepts of time and valence (past-negative/future-positive) was observed, revealing that a mental metaphor can occur between abstract concepts and that multiple metaphors can be processed simultaneously. These results are discussed in terms of different theories within the field of mental metaphors.

## Introduction

Research on conceptualization has demonstrated that participants frequently resort to a metaphorical strategy when processing a concept. This strategy consists of borrowing structural components of a correlating concept to complete the structure of the concept at hand. A good example of this phenomenon is the well-studied mental metaphor^1^ of time onto the sagittal axis of space (past-behind/future-front). This mental metaphor would be a mental transmutation of the experience of time flowing while walking through a path, leaving the past behind and moving forward toward the future (Lakoff and Johnson, 1999). The result, according to Santiago et al. (2011), is a more efficient conceptual representation for the task that is to be carried out with the concept.

Even though the mechanisms involved in this metaphoric process have received much attention during the past decades, many issues are still unresolved or are a matter of debate. This study aims to address three important issues in the field of mental metaphors in order to respond to the following questions: (1) “Can the processing of an abstract concept modulate the processing of another abstract concept?”; (2) “Can multiple mental metaphors be processed at the same time and, if this is the case, what are the underlying mechanisms allowing for this simultaneous activation?”; (3) “Is the focus of attention important for activating a mental metaphor?” Each issue is discussed in detail below.

### Can the processing of an abstract concept modulate the processing of another abstract concept?

Based on linguistic evidence, Lakoff and Johnson (1980) were the first to systematically study mental metaphors, adopting the view that they termed *Conceptual Metaphor Theory*. Their inventory of linguistic expressions reflecting this structural borrowing process was mainly composed of abstract concepts benefiting from the structures of concrete concepts. For example, in the expressions “I am looking *forward* to their response” or “I am feeling *up* today,” the concept of space is used to understand the concepts of time and valence, respectively. On the opposite side, there are no or very few expressions of space being understood by the concepts of time or valence (e.g., you cannot say “I was looking at the plane *positive* in the sky” or “John sat in the *future* of me”). Lakoff and Johnson (1980) concluded that abstract concepts must have a structure that is not as complete as that of concrete concepts due to a lack of grounding in sensory-motor experiences. In order to complete the content of an abstract concept, elements from a more complete conceptual structure (the structure of a concrete concept) would be borrowed.

The results of previous studies investigating the psychological reality of Conceptual Metaphor Theory supported this central assumption. One of the most famous demonstrations was that by Boroditsky (2000), who studied an effect resulting from the mental metaphor of time onto the sagittal spatial axis. Boroditsky (2000) observed that the processing of the concrete domain of space could modulate the processing of the abstract domain of time, but not the other way around. According to her, the frequent use of a mental metaphor would lead to its storage in long-term memory, within the structure of the abstract concept. As a result, the activation of the abstract concept, but not the concrete concept, would also activate the mental metaphor.

However, alternative explanations have been proposed. Walsh (2003) put forward the ATOM theory (A Theory Of Magnitude). According to this theory, all concepts of magnitude (concepts that can be quantified as more or less, such as quantity, space, time or even valence) share neural substrates to process the dimension of magnitude. The simultaneous processing of concepts of magnitude would automatically lead to congruency effects because the different dimensions of magnitude would be processed as a consequence of a shared neural mechanism. A further proposal, the Coherent Working Models (Santiago et al., 2011; for a similar view, see also Marmolejo-Ramos et al., in press), suggests that the mental metaphors are “built” in working memory while the task is carried out. According to the Coherent Working Models, a mental model of the task would be built in working memory to meet task goals. This mental model would include the concepts that are correlating within a task (such as time and space when walking through a path) and form a common representation that is coherent with task goals (past-behind/future-front, for the previous example). After repeated use, a mental metaphor could be stored in long-term memory for further use, but not within the structure of the borrowing concept. This mental metaphor would need to be reactivated in working memory in order to again be part of a mental model. These two alternative explanations have in common the notion that the degree of abstractness (or concreteness) of a concept is no more an issue and they predict bi-directional influences between the concrete and the abstract domains.

Accordingly, Zhong and Liljenquist (2006), Zhong and Leonardelli (2008) and Ouellet et al. (2010a) have demonstrated that processing information from the abstract domain can modulate the processing of the information of the concrete domain. First, Zhong and Liljenquist (2006) studied the link between the purity of the body and morality. Participants were asked to recall an unethical or an ethical deed. They demonstrated that the participants who recalled an unethical deed were more prone to take antiseptic wipes than the participants who recalled an ethical deed. Second, Zhong and Leonardelli (2008) studied the link between the domain of temperature and that of social relationships. People can speak of a “*cold* atmosphere” or a “*warm* person” when speaking about social relationships. In their study, Zhong and Leonardelli (2008) demonstrated the psychological reality of this metaphor by asking their participants to recall a social exclusion or a social inclusion experience. This mere recall was enough to modulate the perception of the temperature in the room. Participants who recalled a social exclusion experience estimated the temperature of the room to be lower than those who recalled a social inclusion experience. Third, Ouellet et al. (2010a) used temporal stimuli to orient participants' spatial attention. They observed a congruency effect according to the left-past/right-future mental metaphor.

Moreover, Santiago et al. (2012) were able to observe a bi-directional conceptual congruency effect for the negative-down/positive-up mental metaphor. In their study, participants were asked to make valence (Experiments 2 and 3) or spatial (Experiments 4 and 7) judgments on positive or negative emotional words presented above or below a face silhouette. When the instructions were simply to make valence (Experiment 2) or spatial (Experiment 4) judgments, they did not observe any congruency effect between the dimensions of valence and space. However, when the participants were endogenously cued to the irrelevant dimension of the task (by asking them to report in which position more words appeared in the valence judgment task or whether there were more positive or negative words in the spatial judgment task), they observed a congruency effect between the dimensions of valence and space in both tasks. Effectively, apart from observing that the concrete spatial domain of space could modulate the processing of valence (Experiment 3), they also observed that the abstract domain of valence could modulate the processing of space (Experiment 7). Thus, the key factor was the attentional focus given to the irrelevant conceptual dimension of the task, rather than the degree of abstractness (or concreteness) of the prime vs. the target.

Another study by Rusconi et al. (2006) also demonstrated that a mental metaphor is possible between two concepts with a low level of abstractness. Effectively, they observed a congruency effect between space and pitch. In their study, when participants were asked to make discrimination judgments on high- and low-pitched sounds with an upper or a lower response key, they were better at making “up” and “down” responses for high-frequency and low-frequencies pitches sounds, respectively, than the opposite.

The last piece missing in order to discard the claim that a mental metaphor necessarily involves an abstract concept benefiting from the more complete structure of a concrete concept is to demonstrate that a mental metaphor can occur between two concepts with a high level of abstractness. To the best of our knowledge, this type of mental metaphor has not yet been demonstrated. From our point of view, the studies that have come closest to achieving this are those showing that the perception of short temporal durations can be modulated by the processing of an abstract concept such as numbers (participants perceive the duration of a stimulus to be shorter when processing low numbers and longer when processing high numbers; see Dormal et al., 2006; Roitman et al., 2007; Vicario et al., 2008). However, a short duration perceived in an online task may not qualify as a concept with a high level of abstractness, due to the fact that the perception of short durations can be directly perceived thanks to the ticking of the participants' internal clock, as discussed by most of the authors of these studies.

In the present study, two concepts with a high level of abstractness were used—time and valence. In order to ensure the abstract nature of the concept of time, verbs conjugated in either the past or future tense were used to refer to these two poles of the time concept (for other studies using a similar strategy, see Torralbo et al., 2006; Ouellet et al., 2010a,b). The second abstract dimension was that of valence (positive or negative). Both dimensions were simultaneously activated due to the use of French positive or negative verbs conjugated in past or future forms (e.g., “haïssaient”—“they hated” or “applaudira”—“he will applaud”). Any interaction between valence and time concepts under these circumstances could only be explained by an interaction between concepts with a high level of abstraction.

Studies on the temporal value asymmetry (TVA) point to the existence of interactions between past-future time and positive-negative valence (e.g., Caruso et al., 2008; Guo et al., 2012). In these studies participants are asked to economically value past or future events. For example, they can be asked how much they would pay for a bottle of wine in order to thank a friend for letting them use his vacation home… 1 week ago or in 1 week from now. Caruso et al. (2008) demonstrated that people assigned a different monetary value to similar events depending on whether they were past or future events. Guo et al. (2012) also demonstrated that this effect was linked to cultural factors; whilst European Canadians gave more value to events in the future than to events in the past, Chinese Canadians showed the opposite pattern. Guo et al. (2012) attributed this cultural effect to the different ways of representing time. In Chinese culture, time is perceived as something cyclic, where the past is central because the future is a simple repetition of what has already happened (Biao, 2001; Guo et al., 2012). In contrast, North Americans have a more linear representation of time in which the future is more important because it is subject to a certain level of control, whereas the past is seen as something that cannot be changed (Graham, 1981; Spears et al., 2001). However, studies on the TVA effect cannot be considered as definitive proof of a congruency effect between two abstract concepts. Effectively, the valuation is made via the use of a concrete concept (money) and the reference to the past or the future is made via reference to episodic events, which are much more concrete than the reference to the past or the future in general. As a consequence, the TVA congruency effect could be explained by an association between the concrete concepts of money and episodic events, rather than an association between the abstract concepts of time and valence.

Even if the findings of Guo et al.'s (2012) study do not constitute direct evidence for the positive and negative valuations of the past and future in a more general and abstract way, it can help to predict the congruency effect that should be obtained with French participants, who also seem to represent time in a linear fashion (Yamada and Kato, 2001). Following this logic, French participants should show a past-negative/future-positive congruency effect. It is also important to note that the task used by Guo et al. (2012) caused the participants to implement a reflective process. Consequently, it is possible that the mechanisms used to value temporal concepts under these task conditions might differ from those (possibly more automatic processes) measured in the mental metaphor literature. For this reason, the task used here was developed to measure more automatic aspects of the time-valence mental metaphor.

### Can multiple mental metaphors be processed at the same time and, if this is the case, what are the underlying mechanisms allowing for this simultaneous activation?

Vicario et al. (2008) studied the simultaneous activation of two mental metaphors: the “short durations—low numbers/long durations—high numbers” and the “short durations—left/long durations—right” mental metaphors. Their findings suggest that at least two mental metaphors can be used at the same time; while participants were making underestimations on the left side and overestimations on the right side they were also making underestimations and overestimations with low and high numbers, respectively. Unfortunately, the analyses conducted by Vicario et al. (2008) did not allow for an assessment of whether these simultaneous activations were independent or whether they interacted together to form a kind of conglomerate representation. Instead of using statistical methods that would have included all the factors (Durations, Space, and Numbers) in a unique design, which allows for confirming the absence or presence of a three-way interaction, they carried out separated analyses for the congruency effects between Durations and Numbers, and between Durations and Space.

It is of importance to examine whether the simultaneous activations of multiple mental metaphors are independent or dependent of each other, since this can provide insights into the nature of the mechanisms that allow for their corresponding congruency effects. As explained above, Boroditsky (2000) considered the possibility that the mental metaphors are stored in long-term memory within the representation of the concepts that benefit from the structural borrowing process. According to her, processing such a concept would automatically activate its associated mental metaphor. In addition, when a mental metaphor is activated, it could not be manipulated. In this case, varying the degree of activation of the conceptual components of a mental metaphor would not have any effect on the processing of that mental metaphor. Thus, processing one mental metaphor that has a particular conceptual component could not modulate the simultaneous processing of another mental metaphor that also has this same conceptual component. For example, if a participant reads the verb “they will succeed”, she/he would activate the “left-negative/right-positive” and the “left-past/right-future” mental metaphors. Both metaphors—activated by this stimulus—would prime the space on the right, but the congruency effect observed for one mental metaphor would not interact with the congruency effect of the other mental metaphor. In other words, the congruency effects would be additive.

Another approach (Santiago et al., 2011; see also Marmolejo-Ramos et al., in press) suggests that the conceptual maps are “built” in working memory while the task is carried out and/or depend on the simultaneous use of the same neural substrates (Walsh, 2003). In both cases (Walsh, 2003; Santiago et al., 2011), the structural components participating in the distinct mental metaphors would be activated and simultaneously used to form these metaphors. Thus, two or more mental metaphors sharing a conceptual domain would also share common resources and compete for the simultaneous use of these resources. As a result, each mental metaphor would modulate the processing of the other, and the congruency effects should therefore interact.

Time and Valence concepts are particularly interesting because they share a common spatial projection on the left-right horizontal axis. On the one hand, the past-left/future-right mental metaphor of time would originate from the correlation between the directional activity of reading/writing and the passage of time during this activity (Ouellet et al., 2010b). On the other hand, Casasanto and Chrysikou (2011), and de la Vega et al. (2013; see also de la Vega et al., 2012, Experiments 2 and 3) demonstrated that the negative-left/positive-right mental metaphor of valence depends on the greater fluency experienced on the dominant side of the body. Consequently, asking participants to make temporal (past or future) or valence (negative or positive) judgments with their left or right hand on negative or positive verbs conjugated to the past or the future would allow the simultaneous activation of three mental metaphors at the same time. Independently of the interacting or independent activation of the mental metaphors, French right-handed participants should show negative-left/positive-right (Valence-Space), past-left/future-right (Time-Space) and past-negative/future-positive (Time-Valence) congruency effects.

### Is the focus of attention important for activating a mental metaphor?

Santiago et al. (2011), based on an extensive review of the literature, concluded that the relevant concepts of a mental metaphor needed to be activated to a certain degree for a congruency effect to happen. It thus follows that attentional factors should play a key role in this conceptual activation. Santiago et al. (2012) were the first to systematically investigate how the focus of attention on the relevant concepts of a mental metaphor could modulate its activation. As explained above, the type of judgment (on valence or vertical space) oriented participants' attention toward the concept that had to be judged and, in order to observe a positive-up/negative-down congruency effect, attention also had to be cued to the irrelevant concept of the task. Another finding is that of Torralbo et al. (2006) who studied a concept (time) that can be mapped onto various conceptual dimensions (sagittal or coronal spatial axis). In their study, participants were asked to judge either verbally or manually (with their left and right hands) the temporal reference of words appearing to the left or right on the screen. The left-past/right-future congruency effect emerged only in the task with manual judgments. The use of the left and right hands cued participants' attention to these spatial locations and allowed the mapping of time onto the left-right horizontal axis. In the same vein, de la Vega et al. (2012) demonstrated that asking right-handed participants to perform a simple lexical judgment task (Experiment 1) on positive and negative words with their left and right hands was not enough to observe a left-negative/right-positive congruency effect. The activation of this mental metaphor was observed only when the attention of the participant was cued on the valence dimension of the stimuli by asking them to make positive or negative judgments on these stimuli (Experiment 2). In their last experiment (Experiment 4), they also demonstrated that attention needed to be cued on the spatial dimension as well by showing that the congruency effect emerged only when the mapping of the spatial response was explicit, not implicit.

Based on these findings, in the present study participants should show greater congruency effects for the mental metaphors that have their conceptual components cued by the task. Because participants had to make temporal and valence judgments (with their left and right hands) in separate blocks, it was expected that the past-left/future-right and negative-left/positive-right congruency effects would be greater in the temporal and valence judgment tasks, respectively. Because the relative salience of the irrelevant conceptual dimension for the past-negative/future-positive congruency effects was similar in both tasks, it was anticipated that this congruency effect would be of a similar size in both tasks.

These predictions run counter to Boroditsky's (2000) and Walsh's (2003) theories. As explained earlier, Boroditsky (2000) assumed that, with time, a mental metaphor becomes a structural component of the borrowing concept. Consequently, the borrowing concept cannot be processed without the activation of its associated mental metaphors. Thus, the degree of attention allocated to the lending concept could not modulate the degree of activation of a mental metaphor. Regarding Walsh's (2003) theory, the activation of the magnitude domain would also be automatic when processing a concept that can be quantified. Thus, attention could not modulate the congruency effects between these concepts.

## Hypotheses

First, based on the findings that mental metaphors can exist between concepts at similar levels of abstractness (Rusconi et al., 2006) and the fact that people from western cultures tend to value more positively future than past events (Caruso et al., 2008; Guo et al., 2012), we expected that the mental metaphor between the abstract dimensions of time and valence would occur so that a past-negative/future-positive congruency effect should emerge. In particular, negative verbs conjugated to past and positive verbs conjugated to the future should be responded to more rapidly and more accurately than negative verbs conjugated to future and positive verbs conjugated to the past, respectively.

Second, we predicted that the mental metaphors between time and valence, time and the coronal axis, and valence and the coronal axis should be activated simultaneously. Looking at whether these simultaneous activations occur independently one of each other or whether they interact will deepen our understanding of the mechanism that allows these simultaneous activations. Independent activations would suggest that mental metaphors are stored in long-term memory, within the structure of the concept that benefits from the lending process (Boroditsky, 2000). These representations would be fixed and could not be manipulated. Interactions between these activations would suggest that mental metaphors are elaborated during the accomplishment of the task. Interactions would occur because the same resources would be used by multiple metaphors at the same time. While the Coherent Working Models (Santiago et al., 2011; see also Marmolejo-Ramos et al., in press) would explain such a phenomenon in terms of the simultaneous use of conceptual information in working memory, the ATOM theory (Walsh, 2003) would suppose that this is due to the shared use of neural substrates to process the domain of magnitude.

Third, based on previous results, we also predicted that the focus of attention should play a role in the activation of the mental metaphors. Because the temporal dimension will be cued in the temporal judgment task (with the left and right hands), the past-left/future-right congruency effect should be greater in the temporal compared to the valence judgment task whilst the converse should be true for the negative-left/positive-right congruency effect. With respect to the past-negative/future-positive congruency effect, we expected this to be of a similar size in both judgment tasks because the salience of the irrelevant dimension in each task was similar. If attention does not modulate the activation of the mental metaphors used here, it would imply that their activation is more automatic than originally hypothesized by Santiago et al. (2011).

## Experiments 1

### Participants

The participants were 32 (28 women; mean age = 18.88 years, *SD* = 1.17) undergraduate students. They were all from the University Blaise Pascal of Clermont-Ferrand (France) and took part in this experiment in exchange for course credit. All participants were French native speakers and reported having normal or corrected to normal vision. They all gave informed written consent according to the Declaration of Helsinki.

### Design

A 2 (Type of judgment: Time vs. Valence) × 2 (Time: Past vs. Future) × 2 (Valence: Negative vs. Positive) × 2 (Response side: Left vs. Right) within-subject design was used.

### Material

The stimuli were 20 positive and 20 negative verbs (see Appendix I for a list of the materials). The positive or negative value of each verb was first determined on the intuition of the experimenters and a post-test with 12 French native speakers unaware of the goals of the present study served to test whether or not these sets of verbs differed significantly in their ratings. The judgment task was programmed in E-Prime and each verb appeared once in a random order at the infinitive form. A Likert scale was used (1: Extremely negative; 2: Very negative; 3: Negative; 4: Neutral; 5: Positive; 6: Very positive; 7: Extremely positive) and, for each verb, the judge had to press one of the keys from 1 to 7. The mean values for the positive and negative verbs were 5.85 (*SE* = 0.28) and 2.38 (*SE* = 0.25), respectively (the mean scores for each verb are presented in Appendix I). This difference was significant in the analyses by participants [*t*1_(11)_ = −30.008, *p* < 0.001] and by items [*t*2_(38)_ = −34.453, *p* < 0.001].

Two lists of 80 stimuli were created. In a first list, half of the verbs were presented inflected to the 3rd singular person of the past and to the 3rd plural person of the future, and the other half was presented to the 3rd plural person of the past and to the 3rd singular person of the future (see Table 1 for examples of the stimuli). The same verbs were presented in a second list, but with the opposite conjugation arrangement. Special care was taken to ensure the consistency of syllable structure, word length, and frequency of the verb lemma across all conditions (see Table 2).

**Table 1:** Examples of the stimuli as a function of their valence, temporal reference, and number.

**Temporal reference**	**Number**	**Valence**	
		**Negative**	**Positive**
Past	Singular	“Trahissait—“she/he betrayed”	“Réussissait”—“she/he succeeded”
	Plural	“Trahissaient”—“they betrayed”	“Réussissaient”—“they succeeded”
Future	Singular	“Trahira”—“she/he will betray”	“Réussira”—“she/he will succeed”
	Plural	“Trahiront”—“they will betray”	“Réussiront”—“they will succeed”

**Table 2:** Means of the principal characteristics of the stimuli (Number of letters, Number of syllables, Frequencies of occurrence in texts and movies data bases) and Standard Errors (in brackets) as a function of the Valence, Temporal reference, and Number of the verbs.

**Temporal reference**	**Number**	**No. of letters and syllables**	**Valence**	
			**Negative**	**Positive**
Past	Singular	No. of letters	9.7(0.4)	10(0.4)
		No. of syllables	3.1(0.1)	3.1(0.2)
	Plural	No. of letters	11.7(0.4)	12(0.4)
		No. of syllables	3.1(0.1)	3.1(0.2)
Future	Singular	No. of letters	7.6(0.3)	7.8(0.3)
		No. of syllables	3.1(0.1)	3.2(0.2)
	Plural	No. of letters	9.6(0.3)	9.8(0.3)
		No. of syllables	3.1(0.1)	3.2(0.2)
Frequencies of occurrences		Texts	69.1(27.3)	86.7(31)
		Movies	46.7(18.3)	64.1(15.1)

*The frequencies of occurrences of the verb lemma are expressed in occurrences per million and are from a texts database and a movies database (New et al., 2004). Results are rounded to the nearest tenth*.

All characters were written in lower case, bold Courier font, point size 18 and presented on a computer screen on a light gray background.

The task was programmed in E-prime and ran on an Intel Core 2 Duo 1.8 GHz computer. The stimuli were presented on a 20-inch LG Flatron screen (6 ms refreshing rate), and an E-prime SRBOX was used to collect participants' responses.

The French version of the hospital anxiety and depression scale (HAD) was used to assess the participants' anxiety and depression levels (Razavi et al., 1989)^2^.

### Procedure and design

Participants sat in a quiet room at approximately 60 cm from the screen. They were first informed that they would have to make either temporal (past or future) or valence (negative or positive) judgments on verbs in different blocks. They were also instructed that they would have to perform these judgments as fast and accurately as possible with their left and right indexes on the left-most and right-most buttons (7.8 cm form center to center) of the SRBOX, respectively.

The first list of verbs was assigned to half of the participants and the second list to the other half. The experiment was divided into two main blocks, each one dedicated to one type of judgment (temporal or valence). Each main block was further divided into two sub-blocks. Within each sub-block, the complete list of verbs was presented. These sub-blocks differed in terms of the mapping of left and right responses for “past” or “future” judgment in the temporal judgments task, and in terms of the mapping of left and right responses for “negative” or “positive” judgments in the valence judgments task. In other words, each participant had to carry out both valence and temporal tasks once with a congruent and once with an incongruent response-key assignment, in counterbalanced order.

The order of presentation of the two types of judgment was counterbalanced across participants, as was the order of presentation of the blocks differing in response mapping, leading to a total of eight different levels of counterbalancing. The complete sequence of the experiment was repeated twice.

**Figure 1:**
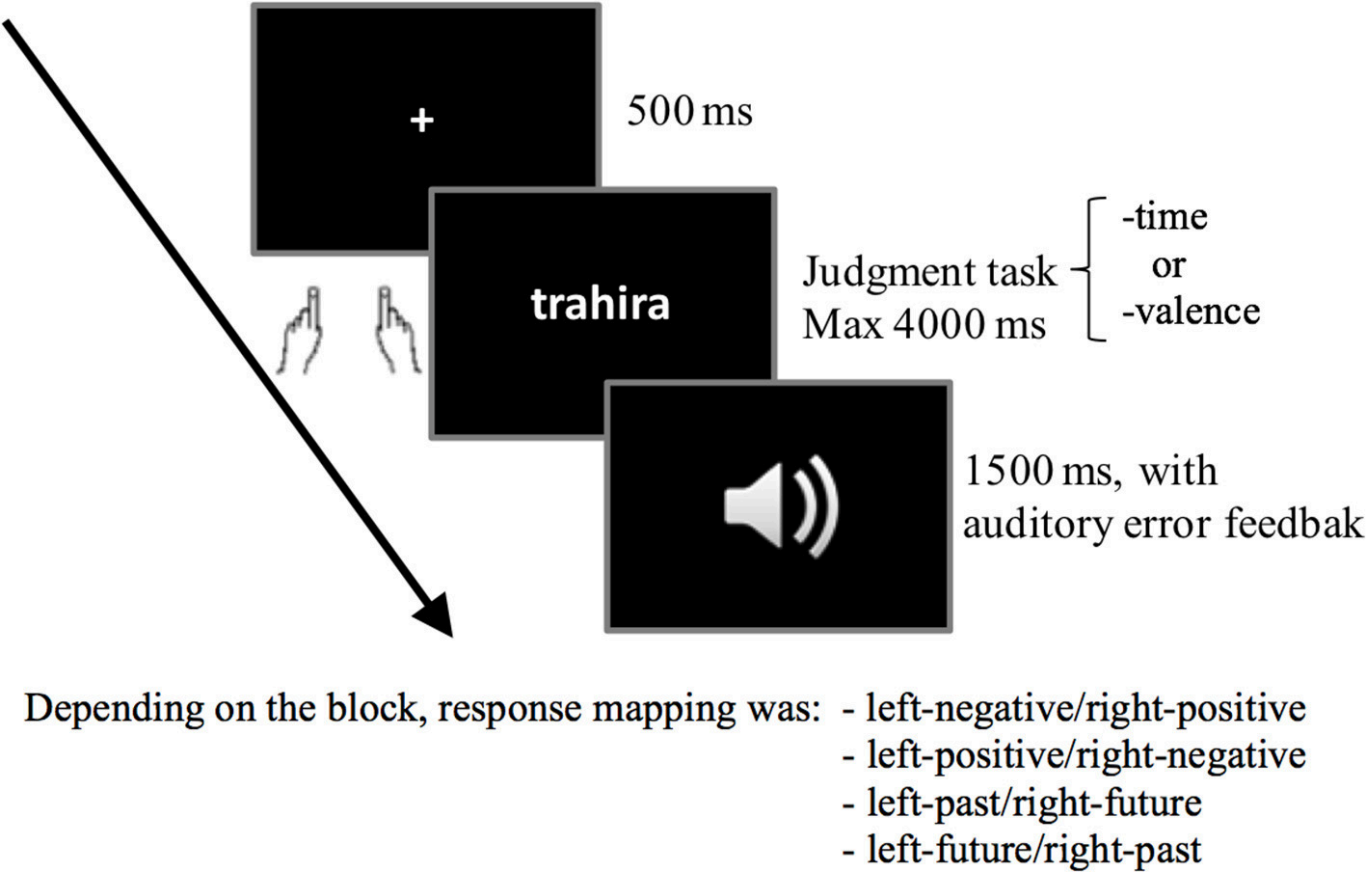
Trial structure in Experiment 1 and Experiment 2. “trahira” means “she/he will betray”.

The trial structure was as follows (see Figure 1). First, a fixation cross (a plus sign) was presented at the center of the screen for 500 ms. The target then appeared for 4,000 ms or until a response was made. Finally, there was a blank screen for 1,500 ms before the next trial. If the participant made an error or did not respond to the target, a BEEP was sounded following the removal of the target.

When the experiment was finished, the French version of the HAD questionnaire was presented to the participant. The participant was instructed to fill it in as honestly as possible and to place it in a box when finished, to ensure anonymity.

### Results

The data set is deposited in a public repository (Spatola et al., 2017). The data from two participants were discarded because they responded randomly in at least one block (around 50% of accurate responses). Responses to two verbs (“vaincre,” meaning “to win,” and “évanouir,” meaning “to faint”) in all conjugated forms were also removed from the analyses because errors were made on more than 25% of the trials in the valence judgment task. The results obtained from the remaining participants and items are summarized in Appendix II. Errors occurred on 3.41% (601 trials) of the trials and these trials were excluded from the reaction times analyses. Correct trials with a reaction time lower or higher than 2.5 SD (standard deviations) per condition for each participant were considered outliers (this filtering procedure has the advantage of taking out extreme values without affecting the data of one condition or of one participant in particular) and also excluded from the reaction time analyses, which corresponded to 583 trials (3.42% of the trials). In order to ensure that the observed effects were not due to the type of filter used, a multiverse analysis was carried out (Steegen et al., 2016). The reaction time filters compared to the one we chose were: 2 median absolute deviations (MAD) and 2 SD per condition for each participant, and no reaction time filter (see Marmolejo-Ramos et al., 2015, for a discussion of the most appropriate reaction time filters according to the characteristics of the data; see also Marmolejo-Ramos et al., 2015). We did not use a logarithmic transformation, because it is problematic when one condition is significantly slower than the other (Lo and Andrews, 2015); judgments of valence were much slower than temporal judgements in the present study. We did not observe any significant interaction between the use of the different filters and the effects of core interest for the present study. The pattern was always similar, but excess data were removed with ±2 SD and ±2 MAD (>5%) and the results were somewhat noisier without any filter.

To assess the effects of Type of judgment (Temporal vs. Valence), Time (Past vs. Future), Valence (negative vs. positive) and Response side (left vs. right) on latency and accuracy data, separate linear mixed models (LMM) were used, one for each dependent variable Brysbaert, 2007; Baayen et al., 2008; Bates et al., 2015). Analyses were run with the lmerTest package for R (Kuznetsova et al., 2016; R Core Team, 2017). Backward elimination of non-significant effects of the linear mixed effect model was performed with the step function. The *p*-values for the fixed effects were calculated based on Sattethwaite's approximation. The model included Type of judgment, Time, Valence and Response side as Fixed effects factors, Subjects and Items as Random effects factors, and the by-subject random slopes for valence (using R syntax, the model tested for the reaction times results was: RT ~ Type_of_judgment ^*^ Time ^*^ Valence ^*^ Response_side + (1+Valence|Subjects) + (1|Items)). The rationale behind the choice of this model and the use of backward elimination can be summarized as follows. In their study, Barr et al. (2013) have shown that the best models were those that use maximal random effect structures justified by the design, followed by the models that use a backward elimination procedure (starting with the maximal structure). Both methods of data analysis have proved to be efficient at avoiding Type I error. However, Bates et al.'s (2015) have demonstrated that Barr et al. (2013) findings were difficult to apply to real data. They demonstrated that maximal random effect structures tend to not converge and that using too many parameters can lead to models that are difficult to interpret. Thus, in order to have a model that is adequate for our experimental design and to avoid Type I error as much as possible, we determined the maximal random effects structure justified by the design before using the backward elimination procedure.

Only the significant or marginally significant findings (with an alpha level < 0.1) corresponding to these models are reported in the text. The results of core interest are summarized in Table 2.

#### Accuracy analyses

**Figure 2:**
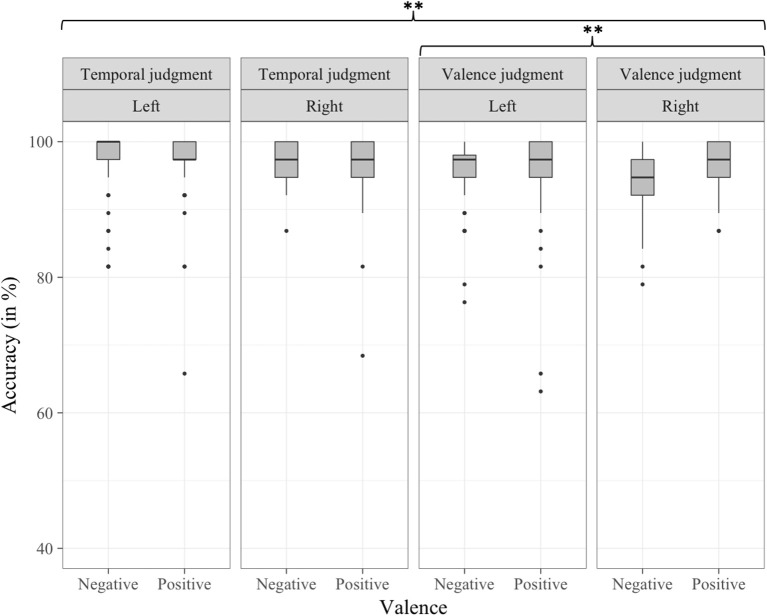
Percentage of correct trials per condition in Experiment 1, as a function of Type of judgment, Valence, and Response side. **p < 0.01.

**Figure 3:**
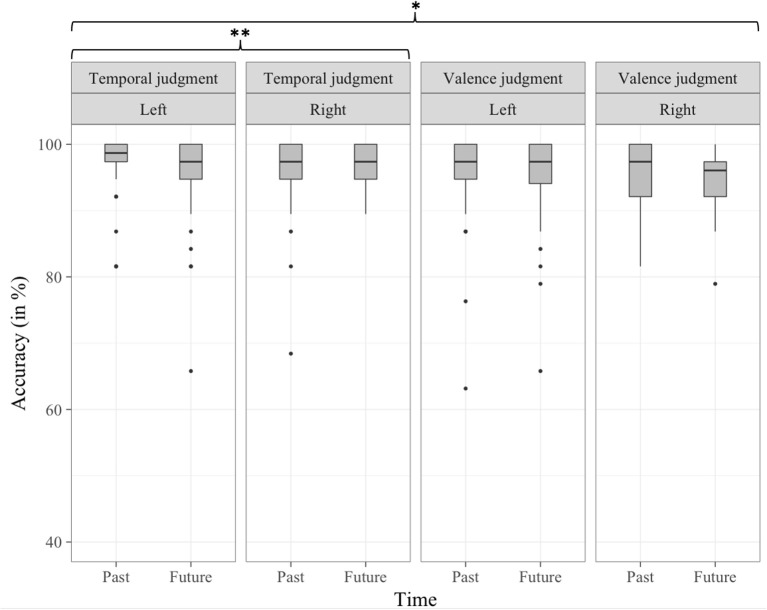
Percentage of correct trials per condition in Experiment 1, as a function of Type of judgment, Time, and Response side. *p < 0.05, **p < 0.01.

Accuracy analyses revealed a significant interaction between Valence and Response side factors [*b* = 2.54, *t*_(9, 014)_ = 3.132, *p* < 0.01]. What is more, this interaction was modulated by the Type of judgment factor [*b* = −3.42, *t*_(9, 014)_ = −2.978, *p* < 0.01; see Figure 2]. The three-way interaction between Type of judgment, Time and Response side was also significant [*b* = 2.46, *t*_(9, 014)_ = 2.138, *p* < 0.05; see Figure 3]. The nature of these three-way interactions was further examined by conducting separate multilevel models on each level of the Type of judgment factor, including both the two-way interactions of Valence by Response side, and Time by Response side. While the interaction between Valence and Response side was significant in valence judgment task [*b* = 2.55, *t*_(4, 460)_ = 2.966, *p* < 0.01], but not in the temporal judgment task [*b* = −0.88, *t*_(4, 460)_ = −1.178, *p* > 0.1], it was the opposite for the Time by Response side interaction, which was significant in the temporal judgment task [*b* = 1.93, *t*_(4, 460)_ = 2.592, *p* < 0.01], but not in the valence judgment task [*b* = −0.53, *t*_(4, 460)_ = −0.614, *p* > 0.1].

Of lesser interest to the purpose of this study, the interaction between Type of judgment and Valence factors was significant [*b* = 3.16, *t*_(9, 014)_ = 3.888, *p* < 0.001], showing that, contrary to the temporal judgment task, participants were making more errors on negative compared to positive verbs in the valence judgment task. The significant interaction between Type of judgment and Time factors [*b* = −1.67, *t*_(9, 014)_ = −2.052, *p* < 0.05] revealed that the greater precision of the participants for past compared to future words was stronger in the valence judgment task than in the temporal judgment task. The main effect of Valence [*b* = −2.15, *t*_(102)_ = −2.664, *p* < 0.01] was also significant: participants were more precise when judging positive compared to negative words.

#### Reaction time analyses

**Figure 4:**
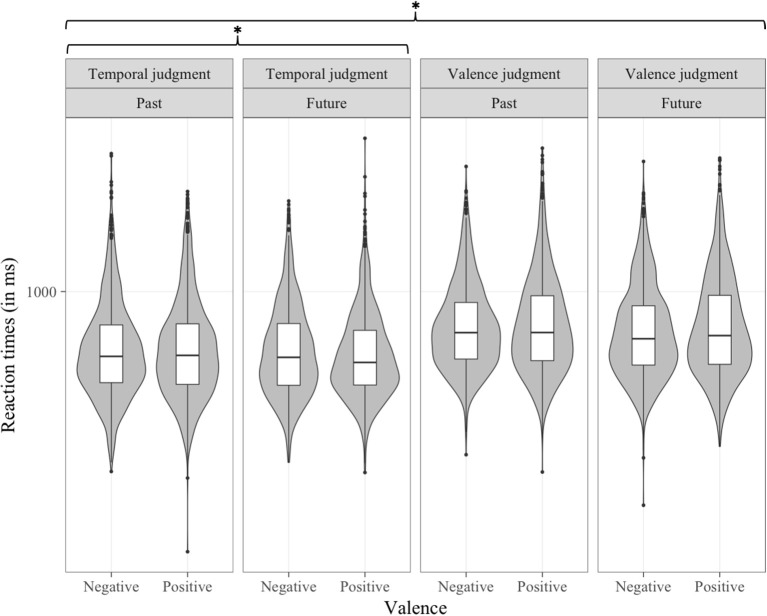
Distribution of RT (in ms) data per condition in Experiment 1, as a function of Type of judgment, Time, and Valence. *p < 0.05.

In the reaction time analysis, the three-way interaction between Type of judgment, Time and Valence was significant [*b* = −39.36, *t*_(8, 980)_ = −2.191, *p* < 0.05; see Figure 4]. A separate multilevel model was conducted on each level of the Type of judgment factor that included the two-way interactions of Time by Valence. These separate multilevel analyses revealed that the negative-past/positive-future congruency effect was significant in the temporal judgment task [*b* = −26.27, *t*_(4, 465)_ = −2.330, *p* < 0.05] but not in the valence judgment task [*b* = 13.6, *t*_(4, 433)_ = 1.061, *p* > 0.1].

Of less interest was the significant Type of judgment by Valence interaction [*b* = 54.88, *t*_(8, 980)_ = −4.321, *p* < 0.001], showing that, while positive verbs elicited faster responses than negative verbs in the temporal judgment task, the opposite was the case in the valence judgment task. The interaction between Type of judgment and Time was marginally significant [*b* = 23.24, *t*_(8, 980)_ = 1.828, *p* = 0.067]: the tendency to respond faster to the verbs conjugated to the future than to the past tended to be greater in temporal judgment task compared with the valence judgment task. The main effect of Type of judgment was significant [*b* = −143.31, *t*_(8, 980)_ = −15.959, *p* < 0.001]: participants were faster on the temporal judgment task compared with the valence judgment task. Participants were also faster to respond to negative compared with positive verbs [*b* = −33.75, *t*_(70)_ = −2.304, *p* < 0.05].

### Discussion

In Experiment 1, a past-negative/future-positive congruency effect was observed, meaning that a mental metaphor can occur between two concepts with a high level of abstractness. This finding goes against the previous belief that the mental metaphors are necessarily used to map the less complete structures of abstract concepts onto the more complete structures of concrete concepts (Lakoff and Johnson, 1980; Boroditsky, 2000). As for the activation of this mental metaphor in the temporal judgment task only, a possibility is that it might be easier to activate this mental metaphor when attention is focused on the dimension of time compared with the dimension of valence. This phenomenon would result from the daily use of this mental metaphor; people speak of the past and the future in negative and positive terms, but they do not speak of the valence dimension in temporal terms. This repeated use of linguistic expressions would result in the storage of the mental metaphor within the conceptual structure of time (for a similar view, see Casasanto, 2008). Thus, processing the dimension of time but not that of valence would activate this mental metaphor. Another possible explanation is related to the intrinsic saliency of the valence vs. the temporal dimensions. In essence, some studies point out the greater saliency of stimuli charged with emotional content compared to other types of stimuli (e.g., Kuchinke et al., 2005; Nakic et al., 2006). If this were the case here, only the concept of valence would be processed in sufficient depth in a secondary task to be able to modulate the processing of other concepts. According to the Coherent Working Models theory (Santiago et al., 2011), a key factor is the intrinsic level of saliency of each dimension. A dimension with a high level of intrinsic saliency processed in secondary task will be processed deeper than a concept with a low level of intrinsic saliency and will more likely be included in a mental model to form a mental metaphor. Accordingly, Santiago et al. (2012) demonstrated that the intrinsic features that contribute to the salience of a conceptual dimension are able to modulate the orientation of exogenous attention. In their study (Experiments 1 and 2), the conceptual dimension of space was processed in the secondary task. The congruency effect between the conceptual domains of valence (processed in the primary task) and space was observed when the salience of the spatial dimension was high (Experiment 1), but not when it was low (Experiment 2). The salience of the spatial dimension was enhanced due to the use of exogenous cues.

In the temporal judgment task, a left-past/right-future congruency effect was also observed, meaning that two mental metaphors can be processed at the same time (see Vicario et al., 2008, for a similar result). Interestingly, since there was no significant three-way interaction between the factors of Time, Valence and Response side, it suggests that the activation of these two congruency effects did not depend on the simultaneous use of shared resources. Thus, processing two (or more) mental metaphors that share conceptual compounds would not contribute to form a conglomerate mental metaphor made up of all these conceptual compounds.

In the valence judgment task only, a left-negative/right-positive congruency effect was observed. This finding, combined with the fact that the past-negative/future-positive and left-past/right-future congruency effects were observed in the temporal task only, demonstrate that the activation of the mental metaphors is context specific and confirm that the orientation of attention is important to activate a mental metaphor (Santiago et al., 2011, 2012).

Despite being interesting, it is important to note that most of the effects were observed in the accuracy results only. It was thus essential to replicate the present results, which was the main goal of Experiment 2.

## Experiment 2

Experiment 2 was almost identical to Experiment 1, with the exception that it was repeated twice and in one of the two sessions a mindfulness-based therapy induction was provided to the participants^3^.

### Participants

The participants were 35 undergraduate students (26 women; mean age = 20.63 years, *SD* = 2.88) from the same population as in Experiment 1. None of them had participated in the first experiment. They all gave informed written consent according to the Declaration of Helsinki.

### Material, procedure, and design

Experiment 2 replicated Experiment 1 in all aspects, with the following exceptions: each participant carried out the complete experimental set described in Experiment 1 twice, once with a mindfulness induction at the beginning of the experimental session and another without any mindfulness induction. Half the participants received the mindfulness induction in the first session and the other half in the second session. One week separated each session.

The mindfulness induction was an audio file^4^ presented to the participant via headphones. The transcript and the translation of the audio file are in Appendix III.

### Results

The data set is deposited in a public repository (Spatola et al., 2017). Three participants were replaced because it was later discovered that they were non-native French speakers. Responses to the two same verbs as in Experiment 1 (“vaincre,” meaning “win” and “évanouir,” meaning “fainting”) in all conjugated forms were also removed. The accuracy and reaction time results obtained from the remaining participants and items are summarized in Appendix IV. Errors occurred on 4.79% of the trials (1849 trials) and these trials were excluded from the reaction times analyses. Following the same filtering procedure as in Experiment 1, correct trials with a reaction time lower or higher than 2.5 SD per condition for each participant were considered outliers and also excluded from the reaction times analyses, which corresponded to 1,138 trials (3.09% of the trials).

The resulting latency and accuracy data were subject to similar analyses as in Experiment 1. The only differences were that the Session and Mindfulness induction were included as Random effect factors in the models, thereby taking into account the variability that they might induce. Only the significant or marginally significant findings (with an alpha level < 0.1) corresponding to these models are reported in the text. The results of core interest are summarized in Table 3.

**Table 3:** Summary of the results of Experiments 1 and 2 that are of core interest for the main goals of this study.

	**Experiment 1**		**Experiment 2**	
**Factors**	**ACC**	**RT**	**ACC**	**RT**
Time × Valence	n.s.	n.s.	^***^	^***^
Time × Response side	n.s.	n.s.	^***^	n.s.
Valence × Response side	^**^	n.s.	^*^	^***^
Type of judgment × Time × Valence	n.s.	^*^	^***^	n.s.
Type of judgment × Time × Response side	^*^	n.s.	n.s.	^***^
Type of judgment × Valence × Response side	^**^	n.s.	n.s.	^***^
Time × Valence × Response side	n.s.	n.s.	n.s.	n.s.
Type of judgment × Time × Valence × Response side	n.s.	n.s.	n.s.	n.s.

* p < 0.05

** p < 0.01

*** *p < 0.001*. *ACC is for accuracy and RT is for reaction time results*.

#### Accuracy analyses

**Figure 5:**
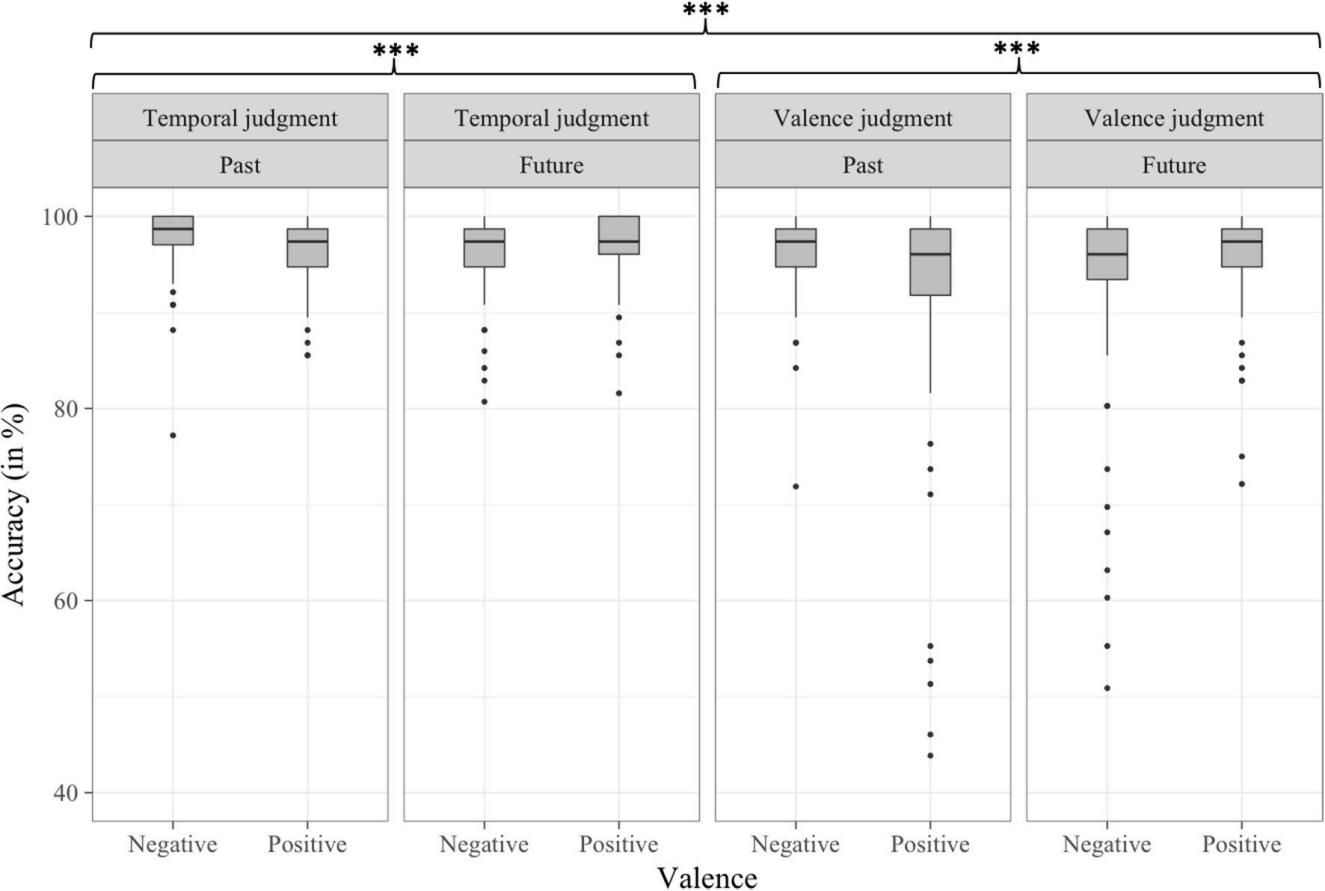
Percentage of correct trials per condition in Experiment 2, as a function of Type of judgment, Time, and Valence. ***p < 0.001.

Accuracy analyses revealed that the Time by Valence interaction was significant [*b* = 8, *t*_(19, 378)_ = 12.585, *p* < 0.001] and this interaction was modulated by the Type of judgment factor [*b* = −6.23, *t*_(19, 378)_ = −6.932, *p* < 0.001; see Figure 5]. One separate multilevel model conducted per level of the Type of judgment revealed that the negative-past/positive-future congruency effect was significant in both the valence [*b* = 8, *t*_(9, 657)_ = 11.352, *p* < 0.001] and the temporal judgment tasks [*b* = 1.77, *t*_(9, 626)_ = 3.36, *p* < 0.001], though the congruency effect was greater in the valence (4%) than in temporal judgment tasks (1%). The Valence by Response side interaction was significant [*b* = 1.1, *t*_(19, 378)_ = 2.448, *p* < 0.05], showing a negative-left/positive-right association. The significant Time by Response side interaction *b* = 2.01, *t*_(19, 378)_ = 4.461, *p* < 0.001] revealed a left-past/right-future congruency effect.

Of lesser interest for the purpose of this study, the Type of judgment by Time interaction was significant *b* = 3.74, *t*_(19, 378)_ = 5.888, *p* < 0.001]: participants were more precise on verbs conjugated to the past compared with the future in the temporal task and it was the opposite in the valence judgment task. The Type of judgment by Valence interaction was significant [*b* = 2.22, *t*_(19, 378)_ = 3.494, *p* < 0.001], showing that participants were more precise when responding to negative compared with positive words in the valence judgment task only. The main effects of Type of judgment [*b* = 1.62, *t*_(19, 378)_ = 3.614, *p* < 0.001], Time [*b* = −5.24, *t*_(19, 378)_ = −10.425, *p* < 0.001], Valence [*b* = −3.57, *t*_(107)_ = 3.614, *p* < 0.001] and Response side [*b* = −1.93, *t*_(19, 378)_ = −4.953, *p* < 0.001] were significant: participants were more precise when judging the verbs' temporal reference, when responding to verbs conjugated to the past, when these verbs were negative or when they responded with their right hand compared with the opposite.

#### Reaction time analyses

**Figure 6:**
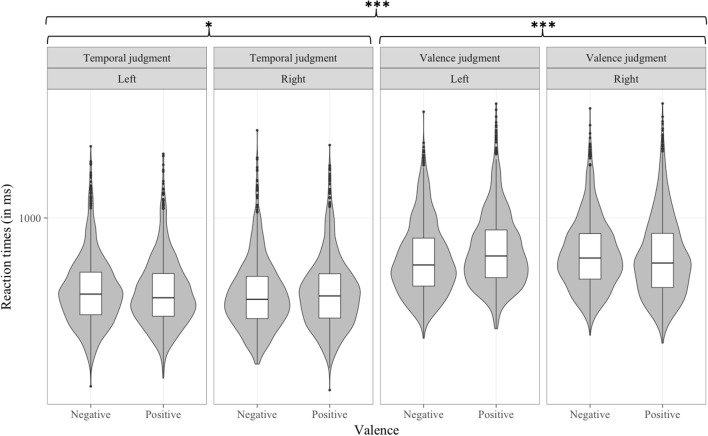
Distribution of RT (in ms) data per condition in Experiment 2, as a function of Type of judgment, Valence, and Response side. *p < 0.05, ***p < 0.001.

**Figure 7:**
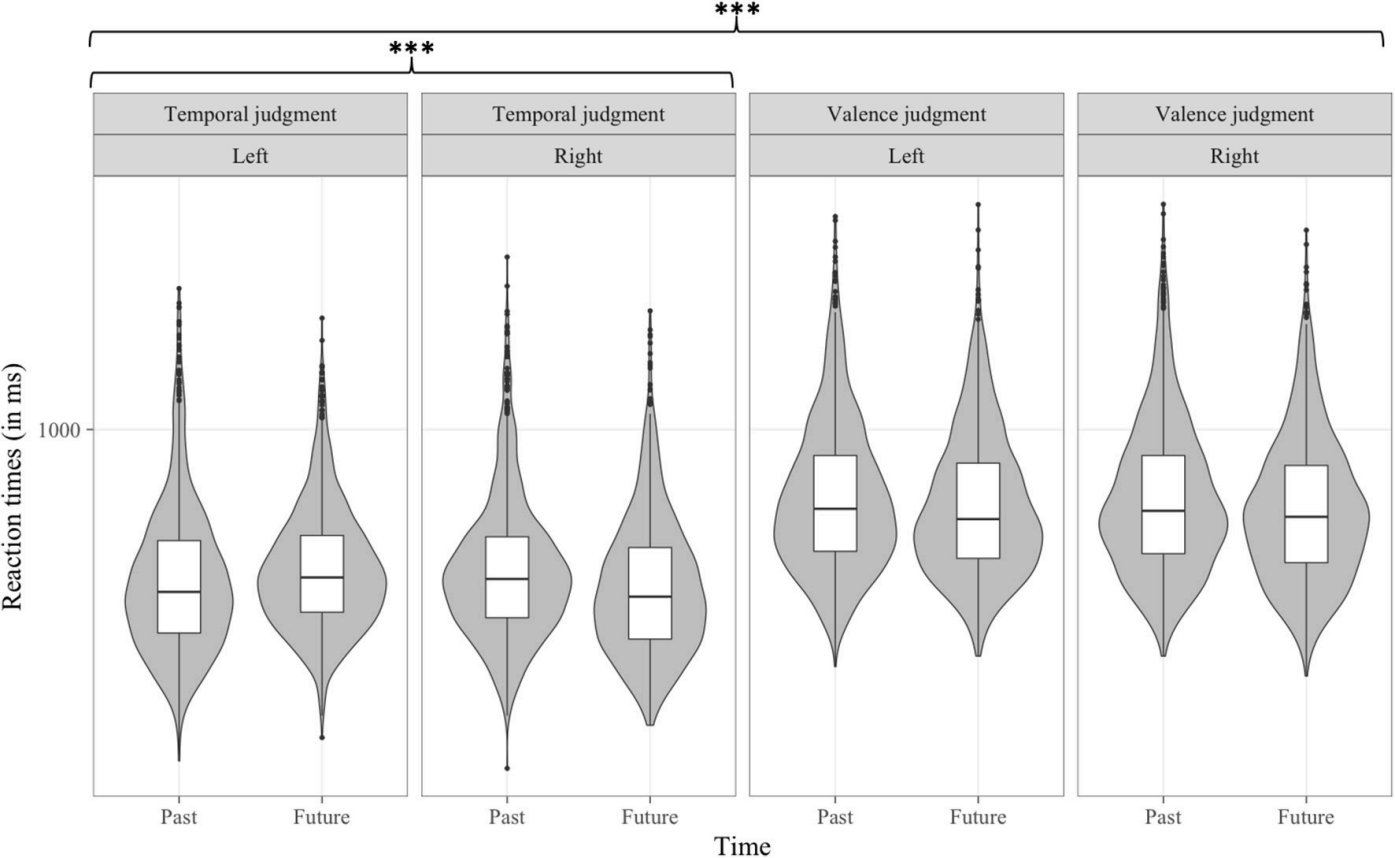
Distribution of RT (in ms) data per condition in Experiment 2, as a function of Type of judgment, Time, and Response side. ***p < 0.001.

In the analysis of reaction times, the Time by Valence interaction was significant [*b* = −24.81, *t*_(18, 937)_ = −4.484, *p* < 0.001], showing a negative-past/positive-future congruency effect. The Valence by Response side interaction was significant [*b* = −57.02, *t*_(18, 938)_ = −7.256, *p* < 0.001]. Further, this interaction was modulated by the Type of judgment factor [*b* = 73.43, *t*_(18, 937)_ = 6.637, *p* < 0.001] (see Figure 6). Separate multilevel models on each level of the Type of judgment factor showed a significant negative-left/positive-right congruency effect in the valence judgment task [*b* = −56.87, *t*_(9, 346)_ = −6.694, *p* < 0.001] and, to our surprize, a smaller (8 ms vs. 29 ms) but opposite congruency effect in the temporal task [*b* = 16.07, *t*_(9, 537)_ = 2.56, *p* < 0.05). The interaction between Type of judgment, Time and Response side was significant, [*b* = −42.54, *t*_(18, 937)_ = −3.845, *p* < 0.001] (see Figure 7). Separate multilevel models on each level of the Type of judgment factor revealed a left-past/right-future association significant in the temporal judgment task [*b* = −50.35, *t*_(9, 538)_ = −8.051, *p* < 0.001] but not in the valence judgment task [*b* = −9, *t*_(9, 371)_ = −1.059, *p* > 0.1].

Of less interest was the significant interaction between Type of judgment and Valence [*b* = −22.01, *t*_(18, 939)_ = −2.816, *p* < 0.01]: in the valence judgement task, responses tended to be faster for the negative than the positive verbs, whilst there was no observable difference in the temporal judgment task. There was a significant main effect of Type of judgment [*b* = −164.07, *t*_(18, 937)_ = −24.296, *p* < 0.001]: participants were faster on the temporal judgment task compared with the valence judgment task. Participants also responded faster to future compared to past verbs [*b* = 41.83, *t*_(18, 941)_ = 6.743, *p* < 0.001]. The main effect of Valence was marginally significant [*b* = 24.04, *t*_(66)_ 1.944, *p* = 0.056], showing a tendency to respond faster to negative than positive words. The main effect of Response side was also significant [*b* = 41.83, *t*_(18, 941)_ = 6.743, *p* < 0.001], revealing that the responses on the right were faster than on the left.

## Discussion

Experiment 2 allowed us to confirm several findings. First, a mental metaphor can occur between two concepts with a high level of abstractness. Second, multiple mental metaphors can be processed at the same time. Third, these simultaneously activated metaphors do not get involved in higher order interactions, and, finally, the orientation of attention is important to activate a mental metaphor.

The observed congruency effects in Experiment 2 were, in general, greater than those observed in Experiment 1 (see Appendix V for a combined analysis of Experiments 1 and 2). We hypothesized that the repetition of the same experiment, which doubled the number of observations, might have reduced experimental noise. Another possibility is linked to the mindfulness induction, which is known to enhance endogenous attention (Ryan and Deci, 2006; Jha et al., 2007; Moore and Malinowski, 2009). If the participant had more attentional resources to process each conceptual dimension and attention plays a key role in the activation of the mental metaphors (Santiago et al., 2011), then the congruency effects should be stronger. However, a further analysis including Mindfulness as a fixed factor did not allow us to observe a pattern in support of this last possibility. Further research will allow us to determine the factors that contribute to a reduction of the experimental noise when processing mental metaphors.

Contrary to Experiment 1, the interaction between the concepts of Time and Valence was observed in both tasks and even greater in the valence judgment task than in the temporal judgment task. The possible reasons for such a discrepancy between Experiment 1 and Experiment 2 are taken up in the General Discussion.

## General discussion

The main findings of the present study can be summarized as follows: (1) a past-negative/future-positive congruency effect was observed only in the temporal judgment task in Experiment 1 and in both tasks in Experiment 2; (2) a left-negative/right-positive congruency effect was observed and this effect was greater in the valence than in the temporal judgment task; (3) a left-past/right-future congruency effect was observed and this effect was greater in the temporal than in the valence judgment task; (4) the three-way interaction between the factors of Time, Valence, and Response side was not significant.

### Can mental metaphors occur between two abstract concepts?

In response to the question “Can mental metaphors occur between two abstract concepts?” the present results indicate that the response is positive. In line with previous results obtained by Guo et al. (2012), a past-negative/future-positive congruency effect was observed in the temporal judgment task in Experiment 1 and in both the temporal and the valence judgment task in Experiment 2. This congruency effect may be linked to the linear representation of time of the French participants (Yamada and Kato, 2001). According to Guo et al. (2012), the cultures that adopt such a representation of time value the future more positively than the past because it is associated with progress and is something that is still under control, whereas the past is, in some ways, of no value because it cannot be changed. According to Guo et al. (2012), again, this congruency effect should reverse in cultures that adopt a cyclical representation of time because the past helps to predict the future and the future is simply a repetition of the past.

This result adds to previous findings, showing that mental metaphors, apart from occurring between an abstract and a concrete concept or between two concrete concepts (Rusconi et al., 2006), can also exist between two abstract concepts. This argues against the assumption made by Lakoff and Johnson (1980), in which the structural borrowing process should necessarily occur from the more complete conceptual structures of the concrete domain toward the less complete conceptual structures of the abstract domain. It is important to note that this congruency effect cannot be attributed to a side effect of the common spatial representations of valence and time. If this were the case, a three-way interaction between the factors of Time, Valence and Response side should have emerged.

The Coherent Working Models (Santiago et al., 2011; see also Marmolejo-Ramos et al., in press) appears to be a better candidate to explain the mental metaphors between abstract concepts. Effectively, this theory claims that, when processing a concept, a mental model is built. This mental model would include information from other concepts in order to form a representation that is more complete for the task at hand. The information included in the model should be coherent with the task that has to be carried out with the concept, and the final result would be a representation that is more effective for that task. According to this view, the degree to which a concept is abstract or concrete would be irrelevant.

A further possible account of these types of mental metaphors emerges from the ATOM theory (Walsh, 2003). Because time and valence can be spoken of in terms of more or less (e.g., “I don't have *much* time” or “I think that Jim is a bett*er* person than Jack”), the magnitude domain of both concepts would be processed by the use of the same neural substrates. This shared use of the “magnitude processor” would result in the congruency effects between both concepts. However, in the task used here, participants were asked to make binary choices (“past”/“future” or “negative”/”positive”), not to judge how much a stimulus was positive or how far in time was the temporal reference of another stimulus. In other words, the domain of magnitude was irrelevant. Further, if that interaction depended on the shared use of a “magnitude processor” and the two other metaphors also depended on the same system, again, a three-way interaction between the factors of Time, Valence, and Response side should have emerged. As a consequence, we consider the Coherent Working Models (Santiago et al., 2011; see also Marmolejo-Ramos et al., in press) to be the best candidate to explain the metaphorical mapping between two abstract concepts.

### Can multiple mental metaphors be processed at the same time and, if this is the case, what are the underlying mechanisms allowing this simultaneous activation?

With regard to the second goal of the present study, the present data suggest that two mental metaphors can be processed at the same time, as in Vicario et al's (2008) study. In Experiment 1, a left-past/right-future and a past-negative/future-positive congruency effects were observed in the temporal judgment task. In Experiment 2, accuracy analyses revealed that the left-past/right-future, left-negative/right-positive and past-negative/future-positive congruency effects were all activated in both the temporal and valence judgment task. Interestingly, the design of the analyses used in this study allowed us to observe that the activations of these mental metaphors were independent from each other. Indeed, the three-way interaction between the factors of Time, Valence, and Response side was not significant. Consequently, this means that the distinct congruency effects observed here did not depend on the simultaneous use of shared resources. If the mental metaphors were *formed* in working memory during the execution of the task (Santiago et al., 2011), each pole of each concept would have been activated in working memory and the information of each pole would have been used to simultaneously form more than one mental metaphor. This competition for the simultaneous use of information corresponding to each conceptual pole would have resulted in a modulation of each of the other mental metaphor processes. The same would have occurred if the mental metaphors were due to the use of shared neural substrates (Walsh, 2003). The multiple mental metaphors would have competed for the use of the shared neural substrates, resulting in a modulation of each of the other mental metaphor processes.

This finding is rather surprising and appears to be more compatible with the Conceptual Metaphor Theory view (Lakoff and Johnson, 1980, 1999; Boroditsky, 2000). However, recent findings strongly suggest that the mental metaphors are processed online, in working memory (see Santiago et al., 2011, for a review). We speculate that when participants form and use mental metaphors, they first keep active in working memory all the poles of each conceptual dimension in order to form the more coherent representations for the task. Then, when these coherent representations are formed, they are processed as a whole in working memory, without the need to keep active their respective poles. Processing the mental metaphors as a whole would reduce cognitive load and would also be in line with attentional models such as that of Corbetta and Shulman (2002). Corbetta and Shulman (2002) hypothesized that the attentional sets that are formed while performing an attentional task are maintained in working memory during the carrying out of that task. Attentional sets and mental metaphors have much in common in that, in both cases, the relevant information for the task is selected and organized to form a coherent representation for carrying out the task. These representations, when kept active as a whole, would produce congruency effects such as those observed here. A side effect would be that processing one mental metaphor would not modulate the processing of another mental metaphor because the mapping information would be available in advance and not *formed* online. More research aimed at testing the time course of the type of information available in working memory while processing mental metaphors is needed to test this hypothesis.

### Is the focus of attention important for activating a mental metaphor?

Regarding the third question about the importance of the orientation of attention to activate a mental metaphor, it was possible to observe that the activation of the mental metaphors was task dependent; the left-negative/right-positive congruency effect was greater in the valence than in temporal judgment task, the left-past/right-future congruency effect was greater in the temporal than in valence judgment task, and a significant past-negative/future-positive congruency effect was observed in the temporal judgment task in Experiments 1 and 2, and in the valence judgment task in Experiment 2. The fact that the left-past/right-future and left-negative/right-positive congruency effects were manifest mainly in the temporal and valence judgment tasks, respectively, is an indication that attentional cueing plays a key role in the activation of the mental metaphors. In this case, participants were given cues by the type of judgment (on valence or time) and the type of response (manual responses with the left or right hand). This finding is congruent with those of de la Vega et al. (2012), Santiago et al. (2012) and Torralbo et al. (2006), who showed that the type of judgment and response could serve as cues for the activation of particular mental metaphors. Interestingly, given that in the opposite task some of the congruency effects could be observed, albeit to a lesser extent, it appears that the effects of attentional orienting on the activation of metaphors is a gradual process. Thus the more attention allocated to the concepts of a mental metaphor, the stronger the activation of this metaphor would be.

Surprisingly, the past-negative/future-positive congruency effect was significant only in the temporal judgment task in Experiment 1 and greater in the valence judgment task in Experiment 2. Here it is important to note that the instructions before starting an experimental block were exactly the same in both experiments and that the irrelevant conceptual dimension (time and valence in the valence and temporal judgment tasks, respectively) was equally cued by the instructions in both judgment tasks (in the temporal task instructions, the words “positive” and “negative” of the valence judgment task were simply interchanged with the words “future” and “past,” respectively). We can think of two possible reasons, one linked to the relative salience of the concepts of time and valence, and the other linked to the activation of different mental metaphors.

According to the relative salience hypothesis, the past-negative/future-positive congruency effect would have been manifest only in the temporal judgment task in Experiment 1 because the conceptual dimension of valence is intrinsically more salient than that of time. Some studies suggest that the dimension of valence is effectively more salient than other more neutral dimensions due to connections with emotional neural structures (e.g., Kuchinke et al., 2005; Nakic et al., 2006). As a consequence, the participants would process the concept of valence more deeply in the temporal judgment task than the concept of time in the valence judgment task. One could argue that, if the dimension of valence was so active in the temporal task in Experiment 1, a left-negative/right-positive congruency effect should also have been observed in the temporal judgment task, which was not the case. Nevertheless, Santiago et al. (2011) argued that mental metaphors are activated in order to create a coherent and efficient representation of the borrowing concept in relation to the task that has to be carried out with that concept. Since the spatial representation of valence is completely unrelated to the temporal judgment task, this mental metaphor would be of no use and not activated in this task. In Experiment 2, the strong temporal component of the mindfulness induction, by asking the participant to focus on the present moment while performing relaxation exercises (see Appendix II) might have driven participants' attention to the temporal dimension, making it more salient. As a result, the concept of time in the valence judgment task would be processed with sufficient depth to modulate the processing of the valence concept.

Another possibility is that the participants of Experiment 1 might have activated a mental metaphor in which the temporal dimension is valued, but not the opposite. Because people frequently value the past and the future but do not temporalize the valence dimension in their daily life, they would come to store this mental metaphor in long term memory along within the structure of time. As a consequence, this would be readily activated when processing the concept of time, but not that of valence (for a similar view, see Casasanto, 2008). As for Experiment 2, the strong temporal component of the mindfulness induction used in a therapy context might have contributed to the temporalization of the dimension of valence, which also led to a past-negative/future-positive congruency effect in the valence task.

The present results do not allow us to distinguish between these two possibilities and these explanations cannot account for the fact that, when Mindfulness was included as a fixed factor in a further analysis, it did not modulate the activation of Time is Valence metaphor in any of the experimental conditions. Further research is needed to directly address this issue. Nevertheless, it is important to note that, no matter what the explanation, these discrepancies between tasks and Experiments highlight the flexibility in the activation of the mental metaphors and this flexibility is predicted by the Coherent Working Models (Santiago et al., 2011).

## Conclusion

The present results allowed us to reveal that it is possible to create a mental metaphor between two abstract concepts, and that the focus of attention is important for activating a specific mental metaphor. Accordingly, the activation of the mental metaphor between the two abstract concepts (time and valence) appeared to depend on attentional factors. These findings are in line with the Coherent Working Models theory (Santiago et al., 2011), but other results are puzzling. In particular, within the state-of-the-art models already published in the scientific literature, it is difficult to explain the independent simultaneous activations of different mental metaphors together with the need for attention to activate a mental metaphor. Here we proposed an adjustment of the Coherent Working Models theory (Santiago et al., 2011), but more research to directly address this issue will be needed before we endorse this possibility. Moreover, we have yet to determine the precise factors explaining why the congruency effect between the dimensions of valence and time was significant only in the temporal judgment task in Experiment 1 whereas it was greater in the valence judgment task in Experiment 2, with a mindfulness induction. It is also unclear why, under certain circumstances, some congruency effects were observed in the accuracy results, but not in the reaction time data (e.g., most of the congruency effects observed in Experiment 1 were only evident in the accuracy data). Future research will therefore be necessary to shed light on the characteristics of the mechanisms responsible for these effects.

## Ethics statement

At the time we carried out these experiments, full review and approval by an ethics committee was not required according to the local and national guidelines. However the LAPSCO lab at University Blaise Pascal had already set up a protocol that adhered to the general principles of the Declaration of Helsinki and we followed it: all participants gave written informed consent, a special care was taken to protect the privacy of the participants and the confidentiality of their personal information, all participants were informed on how to get an appointment with the counseling and psychological services at that same university if they thought they needed it after responding to the HAD questionnaire, and an external person was in charge to receive and process any possible complaint.

## Author contributions

MO led the study. His involvement as that of NS included designing the study, collecting the data, analyzing the data, and writing the manuscript. BB made a significant contribution to the design of the study, he was involved in data collection, and also contributed to data interpretation and manuscript writing. MM, LF and JS made significant contributions to the design of the study, the data analysis and interpretation, and to the writing of the manuscript.

### Conflict of interest statement

The authors declare that the research was conducted in the absence of any commercial or financial relationships that could be construed as a potential conflict of interest.

## Footnotes

^1^In this study, we use the terminology “mental metaphor” as termed by Casasanto (2013) to refer to what Lakoff and Johnson (1980) firstly called “Conceptual Metaphor.” The reasons for this change is that the terminology “conceptual metaphor” refers to mental metaphors being stored within the structure of the borrowing concept, in long term memory, and to a metaphoric process used exclusively to help the processing of abstract concepts, two assumptions that we are rejecting in the current article.

^2^The results corresponding to HAD questionnaire will be analyzed in a further study that will include the results of a greater number of participants in order test whether or not the participants present conceptual biases associated with their mood.

^3^This mindfulness induction was realized in order to test whether or not the biases associated with the mood of the participant can be modulated, but this topic will be analyzed in a further study that will include the results of a greater number of participants in order to run correlation analyses.

^4^Rouget, B. W. (n.d.). Site Francophone sur la Pleine Conscience (Mindfulness) en Psychothérapie. Available online at: http://sites.uclouvain.be/mindfulness/mp3/BW4.mp3. (Accessed October 01, 2013).
