# Supplementary material for: When the Sad Past Is Left: The Mental Metaphors Between Time, Valence, and Space
Source: Front Psychol. 2018 Jun 28;9:1019. doi: 10.3389/fpsyg.2018.01019 (PMC6033299; doi:10.3389/fpsyg.2018.01019)
Supplement: Supplementary file 1 [file Data_Sheet_1.pdf]

Appendix I

Experimental materials

Negative verbs	Scores of the judges	No. of letters and syllables (in brackets)				Frequencies of occurrences	
		Past form		Future form		Texts	Movies
		Singular	Plural	Singular	Plural		
appauvrir (to impoverish)	2.3(0.2)	13(4)	15(4)	10(4)	12(4)	0.56	1.89
bannir (to ban)	2.3(0.4)	10(3)	12(3)	7(3)	9(3)	7.03	3.31
combattre (to fight)	3.4(0.5)	10(3)	12(3)	9(3)	11(3)	42.89	36.35
corrompre (to corrupt)	2.3(0.2)	10(3)	12(3)	9(3)	11(3)	5.32	3.65
craindre (to be scared of)	2.3(0.2)	9(2)	11(2)	8(2)	10(2)	117.44	108.31
décevoir (to disappoint)	2.1(0.2)	8(3)	10(3)	7(3)	9(3)	33.23	21.69
déplaire (to displease)	2.3(0.3)	10(3)	12(3)	8(3)	10(3)	12.23	20.61
détruire (to destroy)	2.8(0.6)	10(3)	12(3)	8(3)	10(3)	126.08	52.36
enlaidir (to become ugly)	2(0.2)	12(4)	14(4)	9(4)	11(4)	0.76	2.36
*évanouir (to faint)	3.2(0.4)	12(4)	14(4)	9(4)	11(4)	18.93	24.93
exclure (to exclude)	2.1(0.2)	8(3)	10(3)	7(3)	9(3)	11.32	16.22
haïr (to hate)	2.3(0.2)	8(3)	10(3)	5(3)	7(3)	55.42	35.68
interdire (to forbid)	2(0.1)	11(4)	13(4)	9(4)	11(4)	63.72	54.39
mentir (to lie)	2.4(0.1)	7(2)	9(2)	7(3)	9(3)	185.16	52.03
perdre (to lose)	2.6(0.1)	7(2)	9(2)	6(2)	8(2)	546.08	377.36
pourrir (to rot)	1.9(0.3)	11(3)	13(3)	8(3)	10(3)	22.86	21.89
punir (to punish)	2.5(0.2)	9(3)	11(3)	6(3)	8(3)	41.7	20.14
salir (to get dirty)	2.5(0.2)	9(3)	11(3)	6(3)	8(3)	17.51	15.95
trahir (to betray)	2(0.2)	10(3)	12(3)	7(3)	9(3)	46.83	41.55
vomir (to vomit)	2.5(0.2)	9(3)	11(3)	6(3)	8(3)	26.12	23.31

Positive verbs	Scores of the judges	No. of letters and syllables (in brackets)				Frequencies of occurrences	
		Past form		Future form		Texts	Movies
		Singular	Plural	Singular	Plural		
applaudir (to applaud)	5.5(0.3)	13(4)	15(4)	10(4)	12(4)	15.82	17.97
attendrir (to touch-emotionally)	5.8(0.2)	13(4)	15(4)	10(4)	12(4)	2.96	20.95
chérir (to cherish)	5.9(0.2)	10(3)	12(3)	7(3)	9(3)	22.66	7.03
détendre (to relax)	5.9(0.2)	9(3)	11(3)	8(3)	10(3)	44.39	23.58
divertir (to amuse)	6.2(0.2)	12(4)	14(4)	9(4)	11(4)	4.49	4.26
épanouir (to blossom)	6.2(0.2)	12(4)	14(4)	9(4)	11(4)	4.51	14.66
fleurir (to bloom)	5.6(0.2)	11(3)	13(3)	8(3)	10(3)	3.95	16.49
garantir (to guarantee)	5.7(0.2)	12(4)	14(4)	9(4)	11(4)	19.74	16.82
jouir (to have an orgasm)	5.8(0.3)	9(2)	11(2)	6(2)	8(2)	22.06	39.19
naître (to be born)	6.1(0.1)	8(2)	10(2)	6(2)	8(2)	116.11	119.32
obtenir (to get)	5.8(0.2)	8(3)	10(3)	9(3)	11(3)	88.08	80.95
offrir (to give)	6.3(0.2)	7(2)	9(2)	7(3)	9(3)	177.8	213.99
plaire (to appeal)	6.2(0.2)	8(2)	10(2)	6(2)	8(2)	607.21	139.8
promettre (to promise)	5.5(0.4)	10(3)	12(3)	9(3)	11(3)	185.26	101.42
recevoir (to receive)	5.8(0.1)	8(3)	10(3)	7(3)	9(3)	192.73	224.46
réussir (to succeed)	5.9(0.2)	11(4)	13(4)	8(4)	10(4)	131.88	122.16
satisfaire (to satisfy)	5.5(0.4)	12(4)	14(4)	9(4)	11(4)	25.1	38.11
séduire (to charm)	5.9(0.3)	9(3)	11(3)	7(3)	9(3)	16.48	22.23
unir (to join)	5.9(0.1)	8(3)	10(3)	5(3)	7(3)	25.15	32.16
*vaincre (to win)	5.8(0.4)	9(2)	11(2)	7(2)	9(2)	27.84	25.68

Note. The verbs are presented in the infinitive form. The frequencies of occurrences of the verb lemma are expressed in occurrences per million and are from a texts database and a movies database (New, Pallier, Brysbaert, & Ferrand, 2004). The Scores of the judges present the mean scores and Standard Error (in brackets) rounded to the nearest tenth of the 12 judges on a Likert scale ranging from 1 (extremely negative) to 7 (extremely positive), the response 4 (neutral) being in the middle. The verbs preceded by an asterisk were not included in the analyses.

Appendix II

Percentage of correct trials and mean latencies (in ms) per condition in Experiment 1, as a function of Type of judgment, Time, Valence, and Response side. Standard Errors are in round brackets and Coefficients of Variance are in square brackets.

Type of Judgment	Response side	Valence	Time	Percentage of correct trials	Mean latencies (in ms)
Temporal	Left	Negative	Past	98 (0.8)[0.04]	727 (28)[0.21]
			Future	96 (1) [0.06]	734 (26)[0.20]
		Positive	Past	97 (0.8)[0.05]	740 (31)[0.23]
			Future	96 (1.2)[0.07]	708 (25)[0.19]
	Right	Negative	Past	97 (0.6)[0.03]	726 (28)[0.21]
			Future	98 (0.5)[0.03]	708 (23)[0.18]
		Positive	Past	96 (1.2)[0.07]	726 (29)[0.22]
			Future	97 (0.5)[0.03]	693 (20)[0.16]
Valence	Left	Negative	Past	96 (0.9)[0.05]	823 (27)[0.18]
			Future	96 (0.9)[0.05]	809 (28)[0.19]
		Positive	Past	96 (1.3)[0.07]	843 (31)[0.20]
			Future	95 (1.3)[0.08]	850 (33)[0.21]
	Right	Negative	Past	95 (0.9)[0.05]	834 (28)[0.19]
			Future	94 (0.8)[0.05]	811 (28)[0.19]
		Positive	Past	97 (0.6)[0.04]	852 (36)[0.23]
			Future	96 (0.7)[0.04]	829 (29)[0.19]

Appendix III

Transcription with the timing (in seconds) of the mindfulness audio file
0 - *Three Ghanta's rings.*
26 - Concentrons-nous sur le moment présent en adoptant une position assise, une posture droite, alerte.
40 - Si nous le souhaitons nous pouvons fermer les yeux.
44 - Et demandons-nous simplement ce qui se passe en nous à cet instant. Quelles sont nos pensées, quels sont nos sentiments, quelles sont nos sensations corporelles ?
65 - Amenons toute notre attention sur ce qui nous habite à cet instant.
73 - Prenons note, simplement, de notre expérience. Juste en cet instant.
97 – Puis, gentiment, dirigeons notre attention vers notre respiration.
107 - En notant chacune de nos inspirations et chacune de nos expirations, se suivant, l'une après l'autre.
124 - Observons, le déroulement naturel de notre respiration sans chercher à l'influencer, à la modifier.
141 – Appuyons-nous sur notre respiration, pour rester présent. Utilisons notre respiration comme point d'ancrage, pour nous ramener dans le moment présent, pour rester dans un état de pleine conscience.
167 - Et enfin, élargissons le champ de notre conscience au-delà de la respiration, cette fois à notre corps, notre corps comme un tout, à notre corps, ici, qui respire, des pieds à la tête.
194 - En prenant conscience aussi de notre posture, droite, éveillée, et des expressions sur notre visage, juste en cet instant.
217 – *Three Ghanta's rings and end of the audio file.*

Translation and the timing (in seconds) of the mindfulness audio file

0 – *Three Ghanta's rings.*
26 - Just concentrate on the present moment by adopting a seated position, with a straight and alert posture.
40 - If you wish, you can close your eyes.
44 - And just ask yourself what is happening to yourself at this moment. What are your thoughts, what are your feelings, what are your bodily sensations?
65 - Jus focus all your attention on what keeps your mind occupied in this moment.
72 - Now, just take note of your experience.
97 – Then, slowly, focus your attention on your breathing.
107 - Note each of your breaths in and each of your breaths out, one by one.
124 - Observe the natural course of your breathing, without seeking to influence it or to change it.
141 – Keep concentrating on your breathing, to remain in the present moment. Use your breathing as an anchor, to bring you back into the present moment, and to remain in a state of full consciousness.
167 – And, finally, broaden your field of consciousness beyond your breathing, to your body now, your body as a whole, to your body, here, breathing, from the head to the feet.
194 - Also be aware of your posture, straight, alert, and the expressions on your face, just at this moment.
217 - Three Ghanta's rings and end of the audio file.

Appendix IV

Percentage of correct trials and mean latencies (in ms) per condition in Experiment 2, as a function of Type of judgment, Time, Valence, and Response side. Standard Errors are in round brackets and Coefficients of Variance are in square brackets.

Type of Judgment	Response side	Valence	Time	Percentage of correct trials	Mean latencies (in ms)
Temporal	Left	Negative	Past	98 (0.4)[0.02]	638 (25)[0.22]
			Future	95 (0.9)[0.06]	660 (19)[0.16]
		Positive	Past	97 (0.6)[0.03]	637 (24)[0.22]
			Future	96 (0.8)[0.04]	647 (16)[0.14]
	Right	Negative	Past	97 (0.8)[0.05]	648 (22)[0.19]
			Future	97 (0.6)[0.04]	622 (21)[0.19]
		Positive	Past	96 (0.7)[0.04]	665 (22)[0.19]
			Future	97 (0.6)[0.03]	621 (20)[0.18]
Valence	Left	Negative	Past	97 (0.6)[0.03]	793 (27)[0.20]
			Future	92 (2.1)[0.13]	791 (24)[0.17]
		Positive	Past	91 (2.4)[0.15]	857 (27)[0.18]
			Future	94 (1.3)[0.08]	814 (24)[0.17]
	Right	Negative	Past	95 (1) [0.06]	821 (24)[0.17]
			Future	93 (1.9)[0.12]	808 (20)[0.14]
		Positive	Past	91 (2.5)[0.15]	827 (29)[0.20]
			Future	97 (0.6)[0.04]	777 (27)[0.19]

Appendix V

Combined analyses of Experiment 1 and Experiment 2

A combined analysis of Experiment 1 and Experiment 2 was run in order to test the differences and similarities between both experiments. The design of the analyses including the data of both experiments was identical to that of Experiment 2, with the following exception: the fixed factor Experiment (Experiment 1 vs. Experiment 2) was added in the models. Only the significant or marginally significant findings (with an alpha level < 0.1) corresponding to these models are reported in the text and only the findings that are of a core interest for this study are commented in details.

Accuracy analyses

Accuracy analyses revealed that the Time by Valence interaction was significant (b = -8.88, t(28400) = -4.701, $p < 0.001$) and this interaction was modulated by the Experiment factor (b = 8.44, t(28400) = 7.795, $p < 0.001$): while the past-negative/future-positive congruency effect was not significant in Experiment 1, it reached significance in Experiment 2 (see Results sections of Experiments 1 and 2). The Time by Valence interaction was also modulated by the Type of judgment factor (b = 8.34, t(28400) = 3.122, $p < 0.01$). A separate multilevel model conducted on each level of the Type of judgment factor that included the two-way interaction of Time by Valence revealed that, though the past-negative/future-positive congruency effect was significant in the temporal (b = 1.4, t(14130) = 3.257, $p < 0.01$) and in the valence judgment tasks (b = 5.31, t(14129) = 9.581, $p < 0.001$), it was greater in the latter (2.67%) than in the former (0.71%). What is more, there was a four-way interaction between the factors Experiment, Type of judgment, Time and Valence (b = -7.28, t(28400) = -4.758, $p < 0.001$). This was due to the fact that the Time by Valence interaction was greater in the valence than in the temporal judgment tasks in Experiment 2 (see results section of Experiment 2), whereas it was not significant in any of the two tasks (all ps > 0.1) in Experiment 1. The Valence by Response side interaction was significant (b = 2.09, t(28400) = -4.134, $p < 0.001$) and this interaction was modulated by the Type of judgment factor (b = -2.14, t(28400) = -3.002, $p < 0.01$). A separate multilevel model conducted on each level of the Type of judgment factor that included the two-way interaction between Valence and Response side revealed that this interaction was significant in the valence judgment task (b = 2.54, t(4462) = 2.965, $p < 0.01$), but not in the temporal judgment task (b = -0,87, t(4462) = -1.178, $p > 0.1$). The Time by Response side interaction was significant (b = 1.59, t(28400) = 4.454, $p < 0.001$), showing a left-past/right-future congruency effect.

Of lesser interest for the purpose of this study were the significant Experiment by Type of Judgment by Time interaction (b = 4.71, t(28400) = 4.349, $p < 0.001$), Experiment by Valence interaction (b = 2.36, t(921) = -3.011, $p < 0.01$), Experiment by Time interaction (b = -5.11, t(28400) = -6.681, $p <$ 0.001), Type of judgment by Time interaction (b = -5.67, t(28400) = -3.005, $p < 0.01$) and Type of judgment by Response side interaction (b = 1.51, t(28400) = 2.997, $p < 0.01$).The main effects of Time (b = 5.2, t(28400) = 3.858, $p < 0.001$) and Response side (b = -2.33, t(28400) = -5.853, $p < 0.001$) were also significant.

Reaction time analyses

In the reaction time results, the significant Time by Valence interaction (b = 62.69, t(28101) = 2.506, $p < 0.05$) revealed a past-negative/future-positive congruency effect. This interaction was

modulated by the Experiment factor (b = -48.75, t(28101) = -3.393, $p < 0.001$) due to the fact that it was significant in Experiment 2, but not in Experiment 1 (see the Results sections of Experiments 1 and 2). The Type of judgment by Time by Valence interaction was also significant (b = -97.97, t(28101) = -2.772, $p < 0.01$). Separate multilevel models, one for each level of the Type of judgment factor, showed that the past-negative/future congruency effect was significant in the temporal (b = -18.84, t(13993) = -3.371, $p < 0.001$) and in the valence judgment task (b = -19.5, t(13834) = -2.754, $p <$ 0.01), but was somewhat greater in the valence (11 ms) compared to the temporal task (9 ms). Moreover, the four-way interaction between Experiment, Type of judgment, Time and Valence was significant (b = 58.93, t(28101) = 2.906, $p < 0.01$), which was due to the fact that the Time by Valence interaction was significant only in the temporal judgment task in Experiment 1 (see results section of Experiment 1), whereas there was no significant difference between both tasks in Experiment 2 (see the Results section of Experiment 2). The Experiment by Valence by Response side interaction was significant (b = -44.29, t(28101) = -3.082, $p < 0.01$) as a result of the left-past/right-future congruency effect significant only in Experiment 2 (see Results sections of Experiments 1 and 2). This three-way interaction was further modulated by the Type of judgment factor (b = -44.29, t(28101) = -3.082, $p <$ 0.01): while there was no significant difference between the temporal and valence judgment tasks in Experiment 1 (because the congruency effect between Valence and Response side factors did not emerge in the reaction times results: see Results section of Experiment 1), in Experiment 2, there was a negative-left/positive-right congruency effect in the valence judgment task and an opposite congruency effect in the temporal judgment task , although the former was greater than the latter (see the Results section of Experiment 2). The Experiment by Type of judgment by Time by Response side interaction was significant and this was due to the fact that, in Experiment 1, the past-left/future-right congruency effect simply did not emerge in the reaction times results (see Results section of Experiment 1) while in Experiment 2, the Time by Response side interaction was significant in the temporal judgment but not in the valence judgment task (see Results section of Experiment 2).

Of lesser interest for the purpose of this study, the Experiment by Type of judgment by Valence interaction (b = -80.45, t(28101) = -4.585, $p < 0.001$), the Experiment by Valence interaction (b = 56.08, t(929) = 4.225, $p < 0.001$), the Experiment by Time interaction (b = 28.85, t(28103) = 2.,32, p < 0.05), the Experiment by Response side interaction (b = 25.24, t(28101) = 2.031, $p < 0.05$), the Type of judgment by Valence interaction (b = 129, t(28101) = 4.218, $p < 0.001$), the main effect of Type of judgment (b = -132.21, t(28101) = -5.663, $p < 0.001$) and the main effect of Valence (b = -83.18, t(669) = -3.297, $p < 0.01$) were all significant.
